# Silicosis Among Engineered Stone Workers in Ontario, Canada: A Case Series of 20 Patients

**DOI:** 10.1155/carj/9386297

**Published:** 2026-10-12

**Authors:** Abeer Abdelsayad, Ambrose Lau, Susan M. Tarlo

**Affiliations:** ^1^ Department of Medicine Faculty of Medicine University of Toronto Toronto Ontario Canada utoronto.ca; ^2^ Respiratory Division Toronto Western Hospital University Health Network, and St Michael’s Hospital Toronto Ontario Canada uhn.ca; ^3^ Health Leadership and Clinical Education Program University of British Columbia Vancouver Canada ubc.ca; ^4^ Center for Research Expertise in Occupational Diseases St. Michael’s Hospital Toronto Ontario Canada stmichaelshospital.com; ^5^ Gage Occupational and Environmental Health Unit Dalla Lana School of Public Health University of Toronto Toronto Ontario Canada utoronto.ca

**Keywords:** Canada, engineered stone, occupational lung disease, silicosis

## Abstract

**Background:**

Silicosis is a preventable occupational lung disease caused by inhalation of respirable crystalline silica. Although traditionally associated with mining, it is increasingly recognized among workers who fabricate engineered stone, also referred to as artificial stone, for kitchen and other countertops. In Canada, updated surveillance data are limited, and no published case series have characterized engineered stone–associated silicosis.

**Methods:**

We report a retrospective case series of 20 patients diagnosed with silicosis following occupational exposure to engineered stone in Ontario, Canada, between November 2015 and September 2025. Demographic, occupational, clinical, radiologic, and functional data were extracted from medical records. Continuous variables are reported as median and interquartile range (IQR).

**Results:**

The median age at diagnosis was 52 years (IQR: 41.5–64.5), and all patients were male, predominantly immigrants from Southeast Asia. Median occupational exposure to silica was 19 years (IQR: 10.5–27.5). Although 80% reported respirator use, adherence was inconsistent, and workplace controls were often inadequate. Cough and dyspnea were the most common presenting symptoms (60% each), with a median diagnostic delay of 13.5 months. Imaging showed simple silicosis in 70% and progressive massive fibrosis in 30%. Pulmonary function tests demonstrated predominantly restrictive impairment with reduced diffusion capacity. Outcomes were significant, including 15% mortality, median age at death of 43 years, chronic hypoxia in 35%, and lung transplant referral in 30%.

**Conclusions:**

Engineered stone–associated silicosis in Ontario is characterized by prolonged silica exposure, inadequate workplace protections, delayed diagnosis, and severe disease at presentation. These findings underscore the urgent need for strengthened regulatory measures to protect engineered stone workers: more effective occupational silica exposure controls, systematic respiratory health surveillance of workers at risk, and earlier recognition and diagnosis of silicosis.

## 1. Background

Silicosis is a preventable occupational lung disease caused by inhaling respirable crystalline silica, which results in progressive lung scarring and heightens the risk of respiratory failure, infection, and early death. Although silicosis has traditionally been associated with mining and heavy industry, recent cases are increasingly common among workers in stone cutting and countertop fabrication, particularly those involved in engineered stone [1, 2].

Engineered stone, also called artificial stone, has become increasingly popular in Canada, as in Australia, Europe, and the United States, because of its durability, aesthetic appeal, and relatively low maintenance requirements. However, unlike Australia, Canada currently lacks national surveillance data describing the number of engineered stone fabrication facilities, the size of the exposed workforce, or trends in occupational exposure to respirable crystalline silica. Consequently, the true burden of engineered stone–associated silicosis in Canada remains uncertain [3, 4].

In Canada, population‐level surveillance data up to 2019 [5] have suggested declining overall incidence rates of silicosis, but there are no updated published surveillance data. There are also no published case series characterizing engineered stone–associated silicosis in Canadian workers [5]. This gap in the Canadian literature raises concern that contemporary cases may be underrecognized, misclassified, or incompletely captured within administrative datasets. Given the documented international emergence of engineered stone–related silicosis and the presence of similar industries in Canada, systematic characterization of affected workers is essential to better understand demographic, occupational, clinical, radiologic, and functional patterns in the Canadian context.

This case series aims to describe the demographic, occupational, clinical, radiologic, and functional characteristics of silicosis among 20 exposed workers in Canada engaged in engineered stonework.

## 2. Methods

### 2.1. Study Design and Case Identification

We conducted a retrospective case study of patients diagnosed with silicosis after occupational exposure to silica‐containing engineered stone materials. Cases were identified through the occupational and respiratory clinics of a university teaching hospital in Toronto, Canada, between November 2015 and September 2025. Diagnosis was based on a relevant history of exposure to respirable engineered crystalline silica and radiologic and/or pathologic findings consistent with silicosis. Patients were referred to our tertiary occupational respiratory clinics through routine clinical pathways, including referrals from family physicians, community respirologists, occupational medicine physicians, workers’ compensation referrals (Ontario Workplace Safety and Insurance Board), and other specialists. Referrals were prompted by respiratory symptoms, abnormal chest imaging, pulmonary function abnormalities, or concern regarding occupational exposure to respirable crystalline silica. Cases were identified through routine clinical referral rather than through a formal screening or surveillance program.

### 2.2. Data Collection

Clinical, occupational, and demographic data were extracted from medical records and structured occupational assessments. Imaging findings were obtained from chest computed tomography (CT) reports. Pulmonary function testing (PFT), six‐minute walk distance (6MWD), and oxygen saturation measurements were collected whenever available. Canadian reference values were used for PFTs, without correction for ethnic background [6].

### 2.3. Definitions

Silicosis was diagnosed based on a documented history of occupational silica exposure and radiologic features consistent with silicosis. Progressive massive fibrosis (PMF) was defined as conglomerate fibrotic masses measuring > 1 cm on chest imaging.

### 2.4. Statistical Analysis

Descriptive statistics were utilized. Continuous variables are reported as median and interquartile range (IQR); categorical variables are presented as counts and percentages. Findings are summarized using three predefined result tables.

### 2.5. Research Ethics Review

Approval for this retrospective review was obtained from the University Health Network Research Ethics Review Board.

## 3. Results

A total of 20 patients were assessed, with data collected from November 2015 to September 2025.

### 3.1. Demographic Characteristics (Table 1)

The cohort consisted of 20 male patients with a median age at diagnosis of 52 years (IQR: 41.5–64.5 years). Most patients were immigrants to Canada, predominantly from Southeast Asia. English language proficiency was variable, with some patients requiring interpreter support. Patients resided across different regions of Ontario, with the majority living in the Greater Toronto Area (Table 1).

**TABLE 1:** Demographic characteristics.

Demographic characteristics	Overall (*n* = 20), No. (%)
Age at diagnosis (*n* = 20), median (IQR), y	52 (41.5–64.5)
Male sex (*n* = 20)	20 (100)
Race and ethnicity (*n* = 20)	
Asian (Southeast Asian)	15 (75)
White (European)	5 (25)
Date of arrival to Canada (*n* = 20)	
1970–1979	2 (10)
1980–1989	6 (30)
1990–1999	4 (20)
2000–2009	7 (35)
2010–2019	1 (5)
English proficiency levels (*n* = 20)	
Able to communicate effectively	10 (50)
Requires interpreter	10 (50)
Year of silicosis diagnosis, *n* (%)	
2019 (*n* = 1, 5%)	
2020 (*n* = 1, 5%)	
2021 (*n* = 3, 15%)	
2022 (*n* = 4, 20%)	
2023 (*n* = 4, 20%)	
2024 (*n* = 6, 30%)	
2025 (*n* = 1, 5%)	

### 3.2. Occupational History

Occupational history is presented in Table 2. All patients reported occupational exposure to engineered stone, with a median duration of stone‐related work of 19 years (IQR: 10.5–27.5 years). Many workers had previous exposure to natural stone before transitioning to engineered stone fabrication. Although some workers reported using dust‐control measures and respiratory protection, preventive practices were frequently inconsistent. Workplace factors contributing to silica exposure included inadequate ventilation, inconsistent respirator use, poor mask fit, and limited workplace controls (Table 2).

**TABLE 2:** Occupational history (number of patients: 20).

Occupational history	Overall (*n* = 20), No. (%)
Years of work in the silica industry (*n* = 20), median (IQR),y	19 (10.5–27.5)
Engineered stone fabrication industry(*n* = 20)	
Nature of work	
Stone cutter (*n* = 20)	20 (100)
Dust controls: water suppression methods (20)	10 (50)
Respirator use (16)	16 (80)
Sometimes	14 (87.5)
Always	2 (12.5)
Type of respirator used (*n* = 16)	
N95 respirator/N95 mask	9 (56)
Cartridge respirator with dust filters	5 (31)
Disposable respirator (used during cutting)	1 (6.2)
Simple/cloth/paper masks	1 (6.2)
Ventilation systems (11)	
None/poor	8 (73)
HVAC	2 (18)
Local exhaust ventilation (LEV)	1 (9)

### 3.3. Clinical Characteristics (Table 3)

Patients most commonly presented with cough and dyspnea, with a median interval of 13.5 months from symptom onset to diagnosis. Approximately half of the cohort had a history of tobacco exposure. CT imaging demonstrated a spectrum of disease severity, including simple silicosis and PMF, with upper‐lobe predominance (Figure 1 and 2), and frequent lymphadenopathy. PFT showed impaired lung volumes and gas exchange, with restrictive physiology as the predominant pattern. During follow‐up, three patients died from silicosis, and several developed advanced disease requiring chronic oxygen therapy, antifibrotic therapy, or lung transplantation assessment (Table 3).

**TABLE 3:** Clinical characteristics (number of patients = 20).

Clinical characteristic	No. (%)
Presenting symptoms (*n* = 20)	
Cough	12 (60)
Shortness of breath (dyspnea)	12 (60)
Weight loss	4 (20)
Low‐grade fever	3 (15)
Fatigue	1 (5)
Chest pain	1 (5)
Hemoptysis	1 (5)
Recurrent pneumonia	1 (5)
Time to correct diagnosis, median (IQR), months after onset of symptoms	13.5 (10–18.5)
Tobacco use (*n* = 20)	
Current or former smoker	10 (50)
Current smoker	1 (5)
Former smoker	9 (45)
Pack‐years among tobacco users, median (IQR)	23.5 (10–45)
Imaging (*n* = 20)	
Predominant CT pattern	
Simple silicosis	14 (70)
Progressive massive fibrosis	6 (30)
Lung zone distribution	
Upper	12 (60)
Lower	2 (10)
Diffuse	6 (30)
Lymphadenopathy	
None	2 (10)
Hilar only	3 (15)
Mediastinal only	0 (0)
Hilar and mediastinal	16 (80)
Calcified lymphadenopathy	10 (50)
Bronchiectasis	6 (30)
Emphysema	9 (45)
PFTs (*n* = 20), median (IQR), (%)	
FEV_1_ (% predicted)	59 (45–76)
FVC (% predicted)	60 (52–74)
FEV_1_/FVC (%)	80 (72–88)
TLC (% predicted)	71 (53–87)
DLCO (% predicted)	56 (42–70)
6MWD (meters), median (IQR)	432 (372–490)
Postexertion SpO_2_ (%), median (IQR), (%)	92% (86.5%–93%)
Ventilatory patterns on baseline PFT (*n* = 20)	
Restrictive ventilatory defect	14 (70)
Mixed obstructive–restrictive pattern	4 (20)
Obstructive airway disease	2 (10)
Outcomes (*n* = 20)	
Deceased due to silicosis, including (1) postlung transplant	3 (15)
Time from diagnosis to death, median (IQR), months (3)	30 (18–72)
Age at death, median (IQR), years (3)	43 (32–62)
Hypoxia	7 (35)
On antifibrotic therapy	7 (35)
Referred for lung transplant	6 (30)
Underwent transplant	2 (10)

**FIGURE 1:**
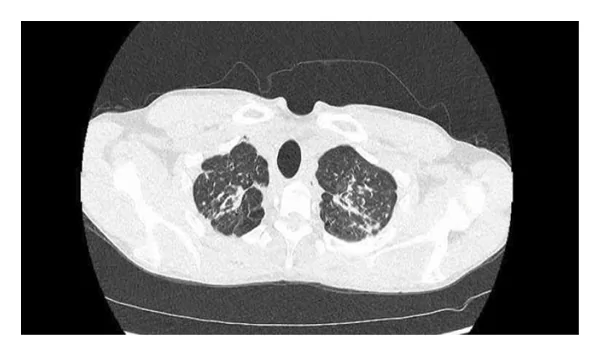
Axial CT image of the chest showing bilateral lung opacities with consolidation and nodularity that have progressed over the course of a year.

**FIGURE 2:**
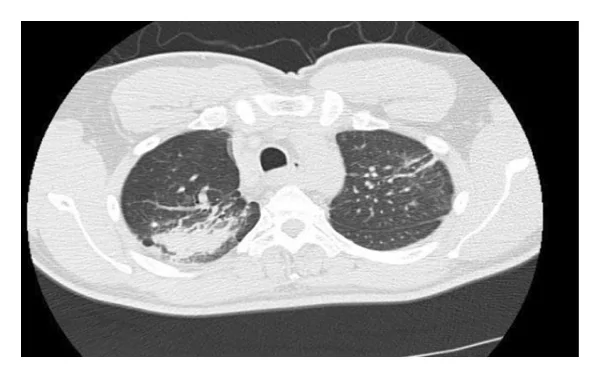
Axial CT chest image demonstrating bilateral upper lobe–predominant reticulonodular opacities with associated fibrotic architectural distortion and mass‐like fibrotic conglomeration, in keeping with progressive massive fibrosis.

## 4. Discussion

This case series presents the demographic, occupational, and clinical characteristics of 20 patients who worked with engineered stone and were diagnosed with silicosis in Ontario, Canada, and includes data collected between November 2015 and September 2025. The findings reveal a pattern of prolonged occupational silica exposure, delayed diagnosis, inadequate workplace controls, and significant disease severity, aligning with emerging reports of re‐emerging silicosis among artificial stone and stone‐cutting workers in Canada and globally.

### 4.1. Demographic and Occupational Correlates

All patients in this cohort were male and primarily immigrants from Southeast Asia and Eastern Europe, reflecting a workforce frequently employed in high‐risk manual occupations such as stone fabrication. International reports of engineered stone–associated silicosis have similarly described predominantly male, immigrant workers employed in small workshops with variable regulatory oversight [1, 3]. In Canada, however, published surveillance data do not yet specifically characterize the demographic profile of workers affected by engineered stone–related silicosis [5]. This gap highlights the importance of describing demographic and occupational patterns within the Canadian context. The demographic pattern of workers in our case series may be influenced by the region from which they were referred.

The median duration of work in the silica industry in this cohort was 19 years. This duration is longer than the exposure periods reported in international outbreaks of engineered stone–associated silicosis, where median or mean exposure durations were typically between 4 and 10 years [4, 7]. Engineered stone products were introduced internationally in the early 2000s, with rapid expansion in Australia, Europe, and North America after approximately 2003–2005. Given this timeline, it is unlikely that the median exposure of 19 years of work with stone in our cohort reflects exclusive exposure to engineered stone. Rather, many workers likely had initial exposure to natural stone and later transitioned to engineered stone or mixed exposures as the use of engineered stone became more widespread in Canada.

Despite the longer cumulative duration of work with stone compared to previous reports, the high rate of PMF (30%), the relatively young median age at diagnosis (52 years), and early mortality indicate a more aggressive disease pattern. This aligns with the severity observed in engineered stone–related silicosis outbreaks in Australia, Spain, and the United States.

### 4.2. Workplace Controls and Respiratory Protection

Despite high self‐reported respirator use (80%), most patients indicated intermittent use, poor mask fit, reuse of disposable respirators, and limited availability of engineering controls. Only half reported water use for dust suppression, and most workplaces lacked effective ventilation systems. These findings align with international evidence that personal protective equipment alone cannot prevent silica exposure when engineering and administrative controls are inadequate [1, 8–10].

Self‐reported compliance may overstate actual protective behaviors, particularly in occupational health clinics, where concerns about job security or employer liability may influence responses. Similar differences between reported and observed respirator use have been documented in Canadian and international occupational hygiene studies [11–14].

### 4.3. Diagnosis, Imaging Surveillance, and Clinical Monitoring

The diagnosis of engineered stone–associated silicosis remains challenging, particularly in early disease, when abnormalities may be subtle. Recent Australian data showed that chest radiography had limited sensitivity for detecting engineered stone–associated silicosis, identifying only 48% of cases confirmed by high‐resolution CT (HRCT) [7]. These findings underscore the limitations of chest radiography alone and support considering low‐dose chest CT in appropriately selected high‐risk workers. Given the subtle nature of early CT abnormalities, surveillance imaging should be interpreted by radiologists with expertise in occupational lung disease to optimize recognition of early silicosis and facilitate accurate follow‐up. These considerations align with updated Australian guidance for physicians assessing workers exposed to respirable crystalline silica dust, which emphasizes appropriate clinical assessment and surveillance strategies [15]. Consistent with recent American Thoracic Society recommendations, prevention should prioritize minimizing exposure, effective engineering controls, appropriate respiratory protection, worker health surveillance, and early recognition and diagnosis of silicosis [16]. Limiting exposure may include avoidance of engineered stone in fabrication when other safer substances can be used.

### 4.4. Clinical Presentation and Diagnostic Delay

Cough and dyspnea were the most common presenting symptoms, consistent with prior silicosis cohorts [8, 17]. The median diagnostic delay of 13.5 months after symptom onset likely contributed to disease progression at presentation. However, asymptomatic disease may have been present well before symptom development, as demonstrated in Australian CT‐based surveillance studies of engineered stone workers, where early radiologic abnormalities were detected in workers without respiratory complaints [8].

In this cohort, many patients were born in Southeast Asia, where prior tuberculosis exposure is more prevalent [18]. Misattribution of upper‐lobe nodularity or fibrotic changes to healed tuberculosis rather than occupational pneumoconiosis may further delay diagnosis, particularly given the known radiologic overlap and increased TB risk in silicosis [12]. In our study, active tuberculosis was excluded through microbiologic evaluation where clinically indicated, and imaging findings were interpreted in conjunction with detailed occupational histories and other clinical features to distinguish silicosis from prior granulomatous disease.

Radiologically, the predominance of upper‐lobe disease, frequent lymphadenopathy, and high rates of calcified nodes are characteristic of silicosis. The observed coexistence of emphysema (45%) and bronchiectasis (30%) likely reflects a combination of smoking exposure, recurrent infection, and silica‐induced airway injury, as described in prior pathological and imaging studies [12, 19–21].

### 4.5. Functional Impairment and Outcomes

PFT revealed predominantly restrictive physiology, with reduced DLCO, consistent with parenchymal fibrosis and impaired gas exchange. The relatively normal FEV_1_/FVC ratio in most patients further supports restrictive or mixed ventilatory issues rather than isolated obstructive disease. The functional impairment was significant, as evidenced by reduced 6MWD and exercise‐induced desaturation, consistent with the disease severity observed in international cohorts of advanced silicosis [22, 23].

The observed mortality rate (15%) and young median age at death (43 years) emphasize the life‐threatening aspect of modern silicosis, even in wealthy countries with access to advanced healthcare. While antifibrotic therapy and lung transplantation are options for some patients, these treatments are expensive, limited in availability, and do not target the underlying causes of the disease [3, 24–26].

### 4.6. Policy, Workplace, and Clinical Implications

These findings have significant implications for occupational health policy and clinical practice in Canada. First, relying solely on respirators without effective engineering and regulatory controls to reduce air levels is insufficient. Consistent enforcement of mandatory wet cutting, local exhaust ventilation, and a ban on dry cutting across all jurisdictions is necessary as a minimum. Second, increased workplace inspections and oversight, particularly in small workshops and subcontracted settings, are crucial. Third, increased regulation to reduce exposure is needed.

From a regulatory perspective, reducing permissible exposure limits (PELs), harmonizing provincial regulations, and enhancing enforcement through provincial ministries of labor in Canada are essential. Occupational exposure limits for respirable crystalline silica vary internationally. Ontario currently applies an 8‐h time‐weighted average exposure limit of 0.1 mg/m^3^ for quartz, compared to the Occupational Safety and Health Administration (OSHA) PEL and the National Institute for Occupational Safety and Health (NIOSH) recommended exposure limit (REL) of 0.05 mg/m^3^, and the American Conference of Governmental Industrial Hygienists (ACGIH) Threshold Limit Value (TLV) of 0.025 mg/m^3^ [27]. Alignment with more protective exposure limits, together with effective implementation and enforcement of exposure control measures, may improve protection of workers in engineered stone fabrication [25, 26]. At a minimum, mandatory baseline and periodic medical surveillance, including spirometry and imaging, should be implemented for high‐risk workers, along with culturally and linguistically appropriate education for workers. However, Australian findings suggest that chest CT scans may be needed to detect asymptomatic disease earlier. Given the subtle nature of early CT abnormalities, surveillance imaging should be interpreted by radiologists with expertise in occupational lung disease to optimize recognition of early silicosis and facilitate accurate longitudinal follow‐up. Due to the practical difficulties in early detection and prevention of engineered stone–associated silicosis, Australia has now banned engineered stone, with the ban coming into effect nationally on July 1, 2024 [28].

Although this study includes only 20 patients, these cases likely represent only a proportion of affected workers in Canada. Experience from other countries demonstrates that clinically recognized cases often represent only a fraction of the underlying disease burden, particularly in the absence of systematic surveillance programs. The growing international recognition of engineered stone–associated silicosis suggests that additional cases may remain undiagnosed among exposed Canadian workers, underscoring the need for improved surveillance strategies and occupational health protections.

Clinically, improved recognition of occupational risk factors, earlier referral to respiratory specialists, and integration of occupational history into routine clinical assessments are necessary to reduce diagnostic delays. Multidisciplinary management, including occupational medicine, respiratory medicine, and social support services, is crucial for affected workers.

## 5. Strengths and Limitations

This case series of contemporary silicosis due to engineered stone in Canada offers detailed clinical, occupational, and functional data from a Canadian context, adding to the limited but growing national literature on contemporary silicosis. To our knowledge, this is the first published case series from Canada, and it describes clinically diagnosed engineered stone–associated silicosis among workers in Ontario. This adds to the growing international evidence, including the initial California case series, which was subsequently followed by identification of 592 cases and major regulatory changes [1]. As a clinically identified case series, our 20 cases may represent only a fraction of the underlying disease burden. Limitations include the small sample size, potential recall bias in self‐reported workplace practices, and referral bias typical of an occupational health clinic population. Nonetheless, the findings align with broader national and international trends and emphasize the urgent need for better preventive measures.

## 6. Conclusion

This case series demonstrates severe but preventable engineered stone–associated silicosis in Canadian workers. These findings support the urgent implementation of effective engineering controls, reduction of respirable crystalline silica exposure, comprehensive medical surveillance, and strengthened occupational health regulations. Further regulatory restrictions, including limitations on the use of engineered stone, should also be considered in Canada, consistent with recent international policy developments.

## Funding

No funding was received for this study.

## Conflicts of Interest

The authors declare no conflicts of interest.

## Data Availability

The data that support the findings of this study are available on request from the corresponding author. The data are not publicly available due to privacy or ethical restrictions.
